# Three polymethoxyflavones from the peel of *Citrus reticulata* “Chachi” inhibits oxidized low-density lipoprotein-induced macrophage-derived foam cell formation

**DOI:** 10.3389/fcvm.2022.924551

**Published:** 2022-07-28

**Authors:** Pu-Lin Liang, Qian-Wen Liang, Pei-Wen He, Xue-Lian Chen, Ya Xu, Hai-Sheng Tu, Liang Zhang, Xiao-Hui Qiu, Jing Zhang, Zhi-Hai Huang, Wen Xu

**Affiliations:** ^1^Key Laboratory of Quality Evaluation of Chinese Medicine of the Guangdong Provincial Medical Products Administration, The Second Clinical College of Guangzhou University of Chinese Medicine, Guangzhou, China; ^2^School of Traditional Chinese Medicine, Guangdong Pharmaceutical University, Guangzhou, China; ^3^State Key Laboratory of Tea Plant Biology and Utilization, Anhui Agricultural University, Hefei, China; ^4^Guangdong Provincial Key Laboratory of Clinical Research on Traditional Chinese Medicine Syndrome, Guangdong Provincial Hospital of Chinese Medicine, Guangzhou, China; ^5^Department of Pharmaceutical Biosciences, Uppsala University, Uppsala, Sweden

**Keywords:** atherosclerosis, polymethoxyflavones, foam cells, inflammation, lipid metabolism

## Abstract

Foam cell formation is the hallmark of the development and progression of atherosclerosis. The aim of this study was to investigate the regulatory effects of three polymethoxyflavones (PMFs), namely, tangeretin (TAN), 5,6,7,3′,4′,5′-hexamethoxyflavone (HxMF), and 3,5,6,7,8,3′,4′-heptamethoxyflavone (HpMF) on macrophage-derived foam cell formation and to further explore the molecular mechanisms. The RAW264.7 macrophage-derived foam cell model was successfully induced by oxidized low-density lipoprotein (ox-LDL) (80 μg/ml). It showed that TAN, HxMF, and HpMF alleviated ox-LDL-induced NO release while also inhibiting the expression of IL-1β, IL-6, and TNF-α in RAW264.7 cells. Uptake of excess ox-LDL was inhibited by TAN, HxMF, and HpMF, resulting in the reduction of its foam cell formation. Moreover, TAN, HxMF, and HpMF promoted HDL-mediated cholesterol efflux. Western blot experiment showed that TAN, HxMF, and HpMF inhibited the expression of scavenger receptor class A type I (SRA1) and cluster of differentiation 36 (CD36), while upregulating peroxisome proliferator-activated receptor γ (PPARγ), liver X receptor α (LXRα), phospholipid ATP-binding cassette transporter G1 (ABCG1), and scavenger receptor class B type I (SRB1) expression. Together, our findings suggested that PMFs inhibited foam cell formation might inhibit lipid uptake *via* downregulating SRA1/CD36 expression and promote cholesterol efflux from foam cells *via* upregulating PPARγ/LXRα/ABCG1/SRB1 expression. This antiatherosclerotic activity is expected to provide new insights into the development of healthcare uses for PMFs.

## Introduction

Atherosclerotic cardiovascular disease (CVD) is the leading cause of CVDs worldwide. Although the pathogenesis of atherosclerosis is not fully elucidated, foam cell retention in the subintima of the arterial wall is considered to be a hallmark of the initiation and development of atheromatous plaque (1–3). Numerous reports indicate that targeting inflammation and lipid metabolism disorders are both major strategies for the prevention and treatment of atherosclerosis (4–6).

Inflammation is involved in the whole process of foam cell formation and atherosclerosis development. Various pathogenic factors stimulate an imbalance in lipid homeostasis within macrophages leading to foam cell formation, while activating various inflammatory processes that accelerate the harmful cycle (7–9). Additionally, the main cause of foam cell formation is related to the excessive accumulation of oxidized low-density lipoprotein (ox-LDL) in macrophages, leading to an imbalance in intracellular cholesterol homeostasis, which involves three processes: lipid uptake, cholesterol esterification, and cholesterol efflux (10). More than 75–90% of ox-LDL internalization in macrophages is mediated by scavenger receptor class A type 1 (SRA1) and cluster of differentiation 36 (CD36), while cholesterol esters in foam cells can be hydrolyzed to free cholesterol, which is then efflux from foam cells mediated by ATP-binding cassette (ABC) transporters and scavenger receptor class B type I (SRB1) in the presence of high-density lipoprotein (HDL) (10, 11). Therefore, promoting cholesterol efflux can impede the development of foam cells. Liver X receptors (LXRs) contribute to the regulation of cholesterol homeostasis in macrophages by positively regulating the expression of phospholipid ATP-binding cassette transporter G1 (ABCG1) (12). Studies have shown that peroxisome proliferator-activated receptor γ (PPARγ) induces ABCG1 and SRB1 expression directly or indirectly *via* stimulation of LXRα expression, further enhancing cholesterol efflux from macrophages. Hence, the PPARγ-LXRα-ABCG1/SRB1 pathway is a potent way to stimulate cholesterol efflux from macrophages. Thus, targeting these pathways is an effective strategy to alleviate foam cell formation.

Adherence to a healthy lifestyle has become a mainstream approach to reducing CVD through primary and secondary prevention, as highlighted in the guidelines (13). Dietary intake of nutrients is one of the most economical and convenient ways. *Citrus* fruits are rich in flavonoid compounds and are an important source of dietary flavonoids. *Citrus* flavonoids have modulating effects on lipid metabolism and oxidative stress, exhibiting potential for the prevention and treatment of atherosclerosis (14–16). Current evidence suggests that fruit flavonoids have therapeutic effects on atherosclerosis through the protection of endothelial cells, inhibition of foam cell formation, regulation of lipid metabolism, and anti-inflammatory pathways (15). The peel of *Citrus reticulata* (Chenpi, CP) and its components are rich in bioactivity and medicinal value. *In vitro* and *in vivo* experiments have shown that CP has protective effects against cardiovascular system diseases and disorders of glucolipid metabolism (17, 18). Guang Chen Pi (GCP), the peel of a specific cultivar *C. reticulata* “Chachi” exclusively collected from the Xinhui district of Guangdong province (19, 20), is considered to be the best CP due to its high medicinal and edible health effects in China. Our previous study showed that polymethoxyflavones (PMFs) are the most influential markers of GCP vs. other types of CP (21, 22). Gao et al. (23) found that PMFs enriched from GCP-ameliorated hyperlipidemia and reduced excessive lipid accumulation and hepatic steatosis in the liver tissues of high-fat diet fed (HFD) mice. Nobiletin, a widely studied hexamethoxyflavone, has been shown to reduce the progression of atherosclerotic disease by preventing dyslipidemia and hepatic steatosis (24, 25). Nobiletin also stimulates the expression of LXRα by activating the AMP-activated protein kinase (AMPK) pathway and further promotes the release of cholesterol from macrophages (26). In addition, the metabolite of nobiletin, 3′,4′-dihydroxy-5,6,7,8-tetramethoxyflavone, reduced LDL oxidation and attenuated the differentiation of monocytes to macrophages and diminished the uptake of modified LDL by macrophages (27). Both tangeretin and heptamethoxyflavone reduced lipid peroxidation (TBARS) in the serum of HFD mice and had a significant therapeutic effect on obesity and hyperlipidemia (28–30). Furthermore, some PMFs from *Citrus* peel regulate lipid homeostasis *via* suppressing multiple scavenger receptors (25, 31–33). These studies indicated that PMFs have a potential role in the prevention and treatment of atherosclerosis.

**FIGURE 1:**
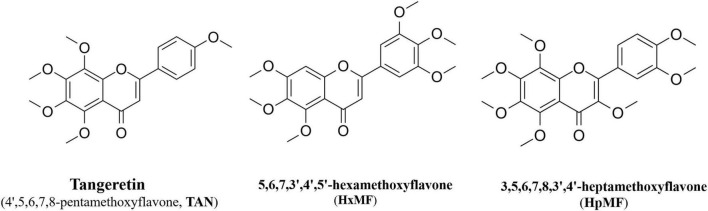
Structures of 4′,5,6,7,8-pentamethoxyflavone (tangeretin, TAN), 5,6,7,3′,4′,5′-hexamethoxyflavone (HxMF), and 3,5,6,7,8,3′,4′-heptamethoxyflavone (HpMF).

In this study, we aimed to investigate the effects of three main PMFs (Figure 1) in GCP, namely, tangeretin (4′,5,6,7,8-pentamethoxyflavone, TAN), 5,6,7,3′,4′,5′-hexamethoxyflavone (HxMF), and 3,5,6,7,8,3′,4′-heptamethoxyflavone (HpMF), on ox-LDL-induced macrophage-derived foam cell formation and inflammation, to further discuss the molecular mechanisms that may be involved in cholesterol homeostasis, and to provide a theoretical basis for the development of plant-based supplements in order to improve cardiovascular health.

## Materials and methods

### Materials and reagents

Dulbecco’s modified eagle medium (DMEM), fetal bovine serum (FBS), phosphate buffered saline (PBS), and fluorescence-labeled cholesterol (22-NBD cholesterol) were purchased from Thermo Fisher Scientific (Waltham, MA, United States) and Oil Red O from Macklin Biochemical (Shanghai, China). The total RNA extraction reagent (TRIzol reagent), Transcriptor First Strand cDNA Synthesis Kit, and FastStart Universal SYBR Green MASTER kit were purchased from Roche (Guangzhou, China). Human ox-LDL, oxidized low-density lipoprotein labeled with 1,1′-dioctadecyl-3,3,3′,3′-tetra-methylindocarbocyanine perchlorate (Dil-ox-LDL), and HDL were supplied by Yiyuan biotechnologies (Guangzhou, China). Briefly, human LDL was purified to homogeneity *via* ultracentrifugation (1.019–1.063 g/cm^3^) and was oxidized using Cu_2_SO_4_ (oxidant) in PBS at 37°C for 18 h. Oxidation is terminated by adding excess EDTA-Na_2_. Each lot is analyzed on agarose gel electrophoresis for migration vs. LDL. This lot of ox-LDL migrates 2-fold further than the native LDL.

### Sample preparation

Dimethyl sulfoxide (DMSO) was used as the vehicle to deliver tangeretin (≥98%, Shanghai Zzbio Co., Ltd., Shanghai, China), 3,5,6,7,8,3′,4′-heptamethoxyflavone (≥99%, Shanghai Zzbio Co., Ltd., Shanghai, China), and 5,6,7,3′,4′,5′-hexamethoxyflavone (Extrasynthese, Lyon, France) to the culture media, which were then diluted to various working concentrations.

### Cell culture and cytotoxicity assay

The murine macrophages RAW264.7 were cultivated in DMEM containing 10% FBS and 100 U/ml of penicillin/streptomycin sulfate in a 5% CO_2_ incubator at 37°C. (3-4,5-dimethylthiazol-2-yl)- 2,5-diphenyl tetrazolium bromide (MTT) assay was performed to detect the effects of 15–250 μM TAN, HxMF, and HpMF on the viability of RAW264.7 cells. Cells viability was finally measured by absorbance at 490 nm using a multifunctional microplate reader (Infinite M1000 PRO, Tecan, Männedorf, Switzerland).

### Anti-inflammatory activity

RAW264.7 cells were seeded into 24-well plates and then stimulated with 80 μg/ml ox-LDL with or without test sample for 24 h. Then, the cell supernatant was collected, and the nitric oxide (NO) secretion was measured with the Griess reagent at 540 nm with a microplate reader. Next, enzyme-linked immunosorbent assay (ELISA) kits (Boster Biological Technology Co., Ltd., Wuhan, China) were used to detect the secretion of interleukin-1 beta (IL-1β), interleukin-6 (IL-6), and tumor necrosis factor-α (TNF-α) in strict compliance with the manufacturers’ instructions.

### Lipid deposition assay

Oil Red O staining was used to observe the phenomenon of the cholesterol esters accumulation in ox-LDL-induced RAW264.7 cells. After planting in 24-well plates, RAW264.7 cells were stimulated by 80 μg/ml ox-LDL with or without three PMFs for 24 h. Then, the cell culture medium was removed, and the cells were washed 3 times with PBS. After fixing with 4% paraformaldehyde for 30 min, the Oil Red O staining was performed for 30 min. Finally, the red lipid drops in RAW264.7 cells were observed using the inverted fluorescence microscope (Nikon, Tokyo, Japan).

After co-culture with PMFs and ox-LDL in 6-well plates, RAW264.7 cells were lysed on ice with RIPA lysis buffer. The triglyceride (TG) and total cholesterol (TC) contents in ox-LDL-induced RAW264.7 cells were measured by the triglyceride assay kit and total cholesterol assay kit (Nanjing Jincheng Biological Engineering, Nanjing, China). Finally, the protein content was measured with the BCA protein assay kit (Beyotime Biotechnology, Shanghai, China) to calibrate the TG and TC contents.

### Oxidized low-density lipoprotein labeled with 1,1′-dioctadecyl-3,3,3′,3′-tetra-methylindocarbocyanine perchlorate uptake assay

For further qualitative and quantitative detection of cellular uptake of ox-LDL, 20 μg/ml Dil-ox-LDL was co-treated with test samples for 3 h. The cells were then washed three times with pre-chilled PBS and then photographed with an inverted fluorescence microscope (Nikon, Tokyo, Japan). In addition, flow cytometry was also used to measure the intake of Dil-ox-LDL by RAW264.7 cells. The calculation of Dil-ox-LDL uptake was based on the geometric mean fluorescence intensity (MFI) of 1 × 10^4^ cells (events).

### Cholesterol efflux assay

A volume of 80 μg/ml ox-LDL was added to stimulate RAW264.7 cells to form foam cells. Simultaneously, 5 μg/ml 22-NBD-labeled cholesterol was added and co-incubated in phenol-free red medium for 24 h. The culture medium was then removed, and the cells were incubated with test samples and 50 μg/ml HDL for 24 h. After incubation, the supernatant was collected, and the cells were lysed with RIPA. Fluorescence intensity of supernatant and lysate was measured by a multifunctional microplate reader (excitation wavelength: 469 nm, emission wavelength: 538 nm). Fluorescence-labeled cholesterol efflux rate = FI supernatant/ (FI supernatant + FI cell lysate) × 100%.

### Real time-quantitative polymerase chain reaction

TRIzol reagent (Invitrogen, Carlsbad, CA, United States) was used to extract total RNA from RAW264.7 cells. The cDNA was then obtained using the Transcriptor First Strand cDNA Synthesis Kit (Roche, Guangzhou, China). Gene amplification was then performed on the ABI Prism TM 7500 (Thermo life, Waltham, MA, United States) using the Roche FastStart Universal SYBR Green Master Kit (Roche, Guangzhou, China). After normalizing the GAPDH housekeeping genes, the relative mRNA expression of each target gene was calculated by the 2^–ΔΔ*Ct*^ method. All primers were synthesized by Sangon Bioengineering Co. (Shanghai, China), and the sequences are shown in Supplementary Table 1.

### Western blot analysis

After treatment with PMFs and ox-LDL for 24 h, RAW264.7 cells were washed with PBS twice and lysed with RIPA on ice for 30 min. The protein concentration was determined by the BCA protein assay kit (Beyotime Biotechnology, Shanghai, China). In total, 40 μg proteins were separated on 8% SDS-PAGE gels and transferred to the polyvinylidene difluoride (PVDF) membrane (250 mA, 90 min). Then, the blots were blocked with 5% bovine serum protein for 1.5 h and incubated with the antibodies overnight at 4°C. The following antibodies were used: SRA1 (1:1,000), CD36 (1:1,000), ABCG1 (1:1,000), PPARγ (1:1,000), and LXRα (1:1,000) were purchased from Affinity Biosciences (Jiangsu, China). SRB1 (1:2,000) was purchased from Abcam (Cambridge, England). GAPDH (1:1,000) was purchased from Cell Signaling Technology Inc. (Boston, MA, United States). Followed by the appropriate HRP-conjugated rabbit anti-rabbit IgG (1:5,000, Cell Signaling Technology Inc., Boston, MA, United States) for 1 h, visualized by the enhanced chemiluminescence (ECL) reagent and exposed to Gel Doc XR+ (Bio-Rad, Hercules, CA, United States). The bands density was analyzed and calculated using the Image Lab software.

### Statistics analysis

All experimental data were expressed as mean ± SD and analyzed using SPSS Statistics 26.0. First, the normality test was performed, and if the distribution was normal, the one-way analysis of variance (ANOVA) in the parametric test was used, and the *F*-test was also performed. If the variances were homogeneous (*p* > 0.05), the differences between groups were judged according to the results of LSD; if the variances were not homogeneous (*p* < 0.05), the differences between groups were evaluated according to the results of Dunnett’s T3. If the normal distribution was not met, the Mann–Whitney *U*-test in the non-parametric test was used to analyze the variance of the samples, and the results were expressed as M (P25, P75). The GraphPad Prism 9 software was used for image plotting. A value of (*) *p* < 0.05 was considered statistically significant, and that of (^**^) *p* < 0.01 was considered highly statistically significant. All experiments were performed independently at three times.

## Results

### Tangeretin, 5,6,7,3′,4′,5′-hexamethoxyflavone, and 3,5,6,7,8,3′,4′-heptamethoxyflavone inhibits oxidized low-density lipoprotein-induced inflammation in RAW264.7 cells

**FIGURE 2:**
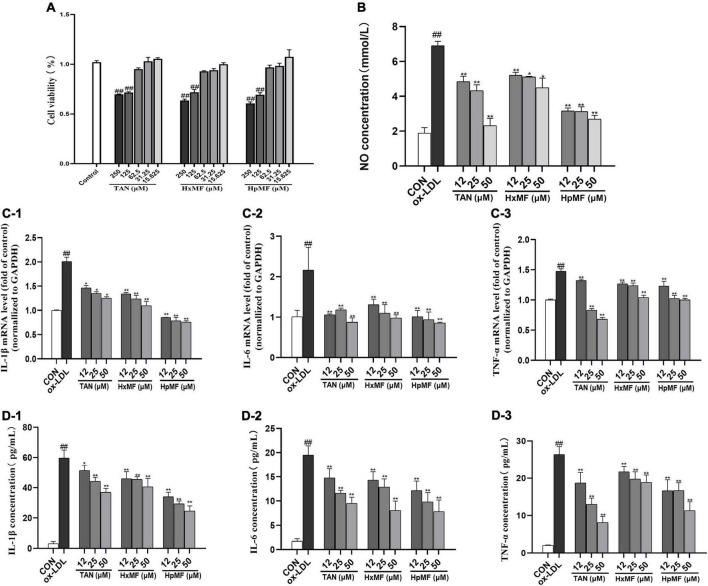
Cytotoxicity of TAN, HxMF, and HpMF at a series of concentrations on RAW264.7 cells **(A)**; effects of TAN, HxMF, and HpMF on ox-LDL-induced production of NO **(B)**; effects of TAN, HxMF, and HpMF on ox-LDL-induced mRNA expression of IL-1β, IL-6, and TNF-α **(C1–C3)**; effects of TAN, HxMF, and HpMF on ox-LDL-induced production of IL-1β, IL-6, and TNF-α in RAW264.7 cells **(D1–D3)**. All experiments were run in triplicate, and data showed mean ± SD. ##*p* < 0.01 compared with the control group, *p < 0.05 and **p < 0.01 compared with the ox-LDL group.

Atherosclerosis is an inflammatory disease in which numerous inflammatory factors and signaling pathways are involved in its development (34). MTT assay showed that TAN, HxMF, and HpMF up to 62.5 μM did not exhibit cytotoxicity to RAW264.7 cells (Figure 2A); therefore, cells were treated with PMFs under the concentration of 50 μM for the follow-up experiments. As shown in Figure 2B, ox-LDL significantly induced the secretion of NO (6.90 mM) compared with the control group (1.88 mM), and all three PMFs significantly inhibited ox-LDL-induced NO accumulation. HpMF showed the highest inhibition of NO secretion, followed by TAN and HxMF.

In RAW264.7 cells, ox-LDL (80 μg/ml) stimulation significantly induced the mRNA expression of the inflammatory factors IL-1β, IL-6, and TNF-α (2.01, 2.16, and 1.48-folds of control, respectively, Figures 2C1–C3). The mRNA expressions of these inflammatory factors were significantly inhibited by TAN, HxMF, and HpMF. Consistently, the three PMFs also effectively inhibited the level of IL-1β, IL-6, and TNF-a in ox-LDL-induced macrophages (Figures 2D1–D3). Notably, 25 and 50 μM TAN exhibited the strongest inhibitory effect on TNF-a expression. Given above, all three PMFs significantly inhibited ox-LDL-induced inflammation.

### Inhibitory effect of foam cell formation

**FIGURE 3:**
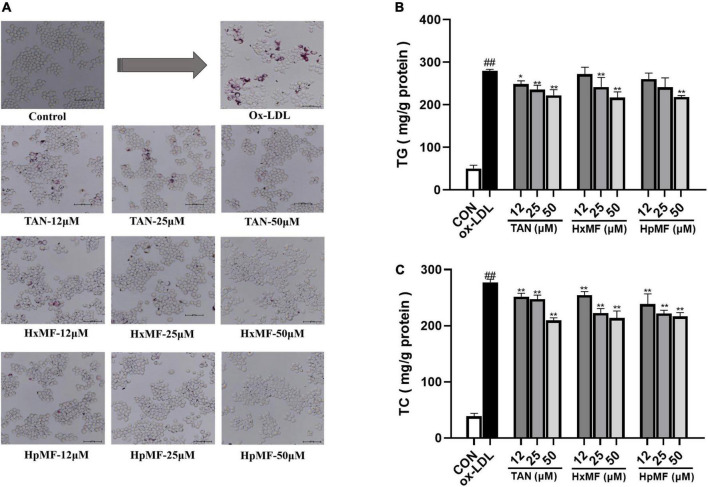
Effects of TAN, HxMF, and HpMF on ox-LDL-induced lipid accumulation by Oil Red O staining **(A)**; effects of TAN, HxMF, and HpMF on triglyceride (TG) **(B)** and total cholesterol (TC) **(C)** contents in ox-LDL-induced RAW264.7 cells. All experiments were run in triplicate, and data showed mean ± SD. ##*p* < 0.01 compared with the control group, *p < 0.05 and **p < 0.01 compared with the ox-LDL group.

Oil Red O staining was used to observe the accumulation of lipids and foam cell formation. As shown in Figure 3A, the number of red lipid droplets was significantly increased, and the lipid droplets were distributed in a ring-like pattern within the ox-LDL-induced RAW264.7 cells, which indicated that the foam cell model was successfully established. Incubation with TAN, HxMF, and HpMF significantly reversed such consequences. In addition, the contents of TG and TC were quantitatively tested. As expected, the levels of TG and TC were significantly increased by ox-LDL stimulation (Figures 3B,C). TAN (12, 25, and 50 μM) significantly reduced TG levels in a dose-dependent manner. The TG content was significantly reduced by HxMF (25 and 50 μM) and HpMF (50 μM) treatments. All three PMFs showed significant inhibition of TC levels in ox-LDL-induced RAW264.7 cells.

**FIGURE 4:**
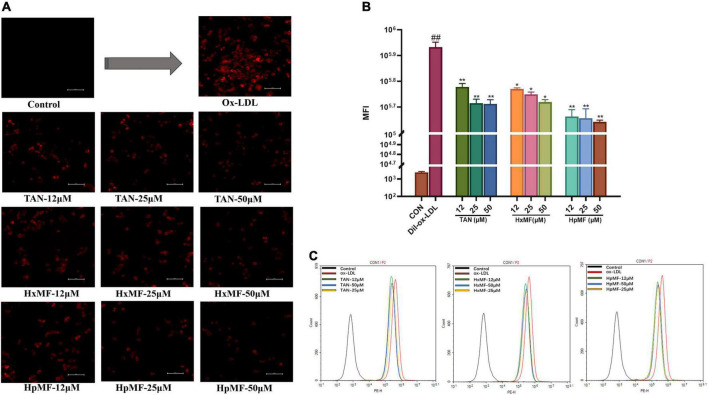
Effects of TAN, HxMF, and HpMF on Dil-ox-LDL uptake by fluorescence microscope **(A)** and flow cytometry **(B,C)**. All experiments were run in triplicate and data showed mean ± SD. ##*p* < 0.01 compared with the control group, *p < 0.05 and **p < 0.01 compared with the ox-LDL group.

Dil-labeled ox-LDL is a popular fluorescent label used to observe cellular uptake of ox-LDL, and it can also quantitatively evaluate the lipid uptake *via* flow cytometry. In our study (Figure 4A), the addition of Dil-ox-LDL (20 μg/ml) significantly enhanced the red fluorescence intensity under the microscope. However, it could be reversed by three PMFs in a dose-dependent manner. These effects were further confirmed by flow cytometry (Figures 4B,C), where HpMF was shown to have the best inhibitory effects on Dil-ox-LDL accumulation.

Overall, we successfully induced the foam cell model by 80 μg/ml ox-LDL and found that TAN, HxMF, and HpMF exhibited significant inhibitory effects on foam cell formation.

### Tangeretin, 5,6,7,3′,4′,5′-hexamethoxyflavone, and 3,5,6,7,8,3′,4′-heptamethoxyflavone inhibits lipid uptake in RAW264.7 cells

To further elucidate the mechanisms by which PMFs affect lipoprotein ingestion by RAW264.7 macrophages, we evaluated the expression of SRA1 and CD36, the two main receptors that regulate lipoprotein internalization. Previous experiments have shown that disruption of the SRA1 or CD36 pathway partially inhibits the uptake of acetylated-LDL (Ac-LDL) or ox-LDL by macrophages while delaying the progression of atherosclerosis in hypercholesterolemic mice (11).

**FIGURE 5:**
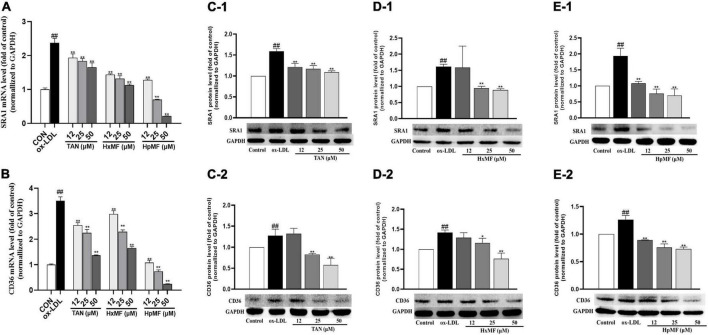
Effects of TAN, HxMF, and HpMF on mRNA expression of SRA1 in ox-LDL-treated RAW264.7 cells **(A)**; effects of TAN, HxMF, and HpMF on mRNA expression of CD36 in ox-LDL-treated RAW264.7 cells **(B)**; effects of TAN on SRA1 and CD36 protein expression in ox- LDL-treated RAW264.7 cells **(C1,C2)**; effects of HxMF on SRA1 and CD36 protein expression in ox-LDL-treated RAW264.7 cells **(D1,D2)**; and effects of HpMF on SRA1 and CD36 protein expression in ox-LDL-treated RAW264.7 cells **(E1,E2)**. All experiments were run in triplicate, and data showed mean ± SD. ##*p* < 0.01 compared with the control group, *p < 0.05 and **p < 0.01 compared with the ox-LDL group.

As shown in Figures 5A,B, the mRNA expression of SRA1 and CD36 induced by ox-LDL were markedly reduced by both TAN, HxMF, and HpMF in the model cell, where HpMF (25 and 50 μM) showed comparable expression levels to the control group. Western blot results showed that HpMF significantly suppressed both SRA1 and CD36 expression from 12 to 50 μM (Figures 5E1,E2), whereas HxMF was only effective above 25 μM (Figures 5D1,D2). TAN markedly inhibited SRA1 expression in the range of 12–50 μM and suppressed CD36 expression at 25 and 50 μM (Figures 5C1,C2). Thus, reduced lipid uptake by three PMFs might be associated with the inhibition of SRA1 and CD36 expression in ox-LDL-induced foam cells.

### Tangeretin, 5,6,7,3′,4′,5′-hexamethoxyflavone, and 3,5,6,7,8,3′,4′-heptamethoxyflavone promotes cholesterol efflux

**FIGURE 6:**
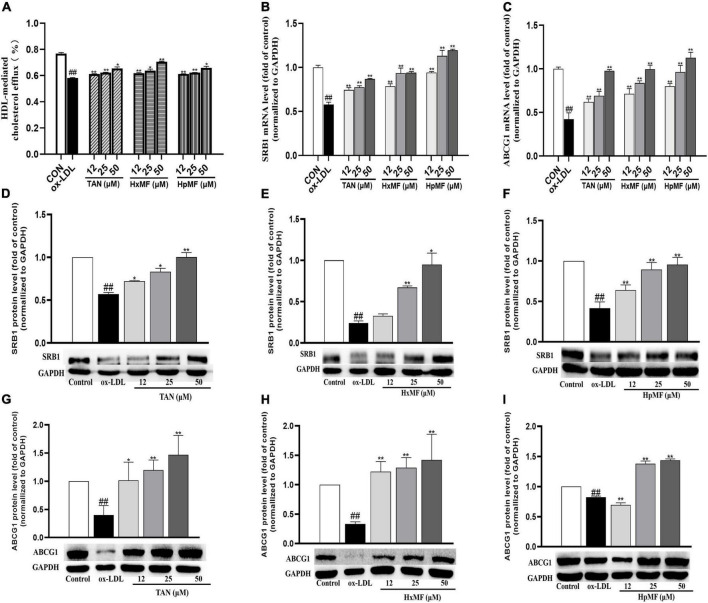
Effects of TAN, HxMF, and HpMF on ox-LDL-induced cholesterol efflux **(A)**; effects of TAN, HxMF, and HpMF on mRNA expression of SRB1 and ABCG1 in ox-LDL-treated RAW264.7 cells **(B,C)**; effects of TAN, HxMF, and HpMF on protein expression of SRB1 in ox-LDL-treated RAW264.7 cells **(D–F)**; and effects of TAN, HxMF, and HpMF on protein expression of ABCG1 in ox-LDL-treated RAW264.7 cells **(G–I)**. All experiments were run in triplicate and data showed mean ± SD. ##*p* < 0.01 compared with the control group, *p < 0.05 and **p < 0.01 compared with the ox-LDL group.

Cholesterol efflux is another important process that maintains lipid homeostasis in macrophages. A fluorescence-labeled cholesterol (22-NBD cholesterol) was used to observe HDL-mediated cholesterol efflux from ox-LDL-induced RAW264.7 cells. As shown in Figure 6A, stimulation with ox-LDL dramatically reduced the cholesterol efflux rate in macrophages (58.23%) compared with control group (76.76%). Both TAN, HxMF, and HpMF significantly improved the efflux rate in a dose-dependent manner.

The molecular mechanisms of their inhibitory effects on cholesterol efflux from foam cells were further explored. As shown in Figures 6B,C, ox-LDL caused the significant decrease in SRB1 and ABCG1 mRNA expression (0.58 and 0.42-folds of control, respectively), and all three PMFs significantly reversed it in a dose-dependent manner. As shown in Figures 6D–F, Western blot results showed that SRB1 protein expression was significantly increased by TAN (12, 25, and 50 μM), HxMF (25 and 50 μM), and HpMF (12, 25, and 50 μM). In addition, ABCG1 protein expression was dose-dependently increased by TAN and HxMF (Figures 6G,H). Although low concentrations of HpMF had no regulatory effect on ABCG1 protein expression, HpMF significantly boosted ABCG1 levels at 25 and 50 μM (Figure 6I).

**FIGURE 7:**
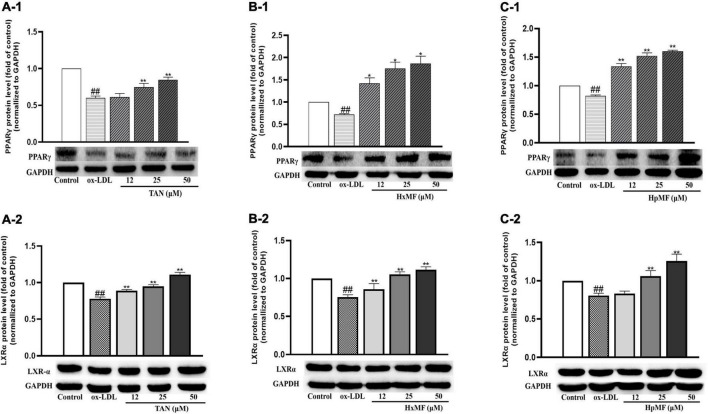
Effects of TAN on protein expression of PPARγ and LXRα in ox-LDL-treated RAW264.7 cells **(A1,A2)**; effects of HxMF on protein expression of PPARγ and LXRα in ox-LDL-treated RAW264.7 cells **(B1,B2)**; effects of HpMF on protein expression of PPARγ and LXRα in ox-LDL-treated RAW264.7 cells **(C1,C2)**. All experiments were run in triplicate and data showed mean ± SD. ##*p* < 0.01 compared with the control group, *p < 0.05 and **p < 0.01 compared with the ox-LDL group.

Liver X receptor α (LXRα) is a target gene of PPARγ that directly or indirectly regulates the expression of SRB1 and ABCG1 in macrophages and promotes cholesterol efflux (35, 36). We were interested in exploring whether PMFs might regulate PPARγ and LXRα to promote ox-LDL-induced cholesterol efflux from foam cells. As shown in Figures 7A1–C1, PPARγ protein expression was significantly increased by TAN (25 and 50 μM), HxMF (25 and 50 μM), and HpMF (12, 25, and 50 μM). Furthermore, TAN and HxMF exposure to 12, 25, and 50 μM also significantly increased LXRα protein expression in ox-LDL-induced RAW264.7 cells (Figures 7A2,B2). Similar to the regulation of ABCG1 protein expression, HpMF did not exhibit a regulatory effect on LXRα at 12 μM, with a significant elevated effect when exposed to 25 and 50 μM (Figure 7C2). All these results demonstrated that TAN, HxMF, and HpMF might promote cholesterol efflux through the PPARγ-LXRα-ABCG1/SRB1 pathway in ox-LDL-induced foam cells.

## Discussion

Flavonoids, a common and naturally occurring phytonutrient with a wide range of biological activities, are the main chemical constituents in CP (14). Unlike other flavonoids with low bioavailability in the human body, PMFs with their hydroxyl groups covered by methylation have a great advantage in terms of oral bioavailability (37). Given that CP and PMFs exhibit significant inhibitory effects on different animal models of atherogenic risk factors, such as hyperlipidemia, obesity, and inflammation (29, 38, 39), this study further investigated the effects of three main PMFs from the peel of *C. reticulata* “Chachi” on the development of atherosclerosis from the perspective of inflammation and foam cell formation.

Inflammation is one of the initiating stages in the development of atherosclerosis. Macrophage uptake ox-LDL and transform to foam cells, while increasing the expression of inflammatory factors and mediators (5). Previous studies have found that PMFs with higher methoxy content have a stronger inhibitory effect on LPS-induced NO secretion in RAW264.7 macrophages (40). Similar findings were confirmed in our experiments, where HpMF exhibited the best inhibitory effect on ox-LDL-induced NO production and mRNA expression of inflammatory factors, i.e., IL-1β and IL-6 (Figures 2C1,C2). The enhanced inhibitory effect of HpMF on ox-LDL-induced inflammation may be related to its methoxy content.

The marked inhibitory effect of three PMFs on ox-LDL-induced foam cell formation was confirmed by the combination of Oil red O staining, Dil-ox-LDL uptake, and lipid content measurements. The inhibitory effect of PMFs on foam cell formation was also manifested in terms of both lipid uptake and cholesterol efflux. Reduced ox-LDL internalization by TAN, HxMF, and HpMF might be associated with their remarkable repression of SRA1 and CD36 expression at both the transcriptional and translational levels. Additionally, PMFs’ promotive effect on HDL-mediated cholesterol efflux might be related to the significant upregulation of SRB1 and ABCG1, as well as the upstream genes PPARγ and LXRα. This suggested that PMFs might induce LXRα expression *via* activating PPARγ and thus upregulate SRB1 and ABCG1 protein expression.

Referring to inhibiting lipid uptake and promoting cholesterol efflux, it seems that HpMF shows a better effect. We speculate it is related to its higher number of methoxy. A study on HepG2 cells suggested that the all-methoxy A-ring of citrus flavonoids may be the most effective structure for the regulation of hepatic lipid metabolism, which can reduce cholesterol and TG concentrations in HepG2 cells by inhibiting the secretion of hepatic apolipoprotein B (41). Similarly, the increase in the number of methoxy on the A-ring of PMFs enhanced the activity of leukemic HL60 cells, while an increase in the number on the B-ring reduced the activity of PMFs on HL60 cells (42). The structure–function relationships of the PMFs need further experimental validation.

In conclusion, our study demonstrated that three main PMFs from the peel of *C. reticulata* “Chachi,” namely, TAN, HxMF, and HpMF significantly inhibited ox-LDL-induced inflammatory factor secretion. PMFs inhibit foam cell formation, might inhibit lipid uptake *via* downregulating SRA1/CD36 expression, and promote cholesterol efflux from foam cells *via* upregulating PPARγ/LXRα/ABCG1/SRB1 expression. This finding is expected to provide a theoretical basis for the development of bioactive PMFs as plant-based supplements.

## Data availability statement

The raw data supporting the conclusions of this article will be made available by the authors, without undue reservation.

## Ethics statement

Ethical review and approval was not required for this study in accordance with the local legislation and institutional requirements.

## Author contributions

P-LL: investigation, formal analysis, data curation, writing—original draft, and methodology. Q-WL: formal analysis and data curation. P-WH and YX: investigation and data curation. X-LC and H-ST: investigation. LZ and X-HQ: conceptualization and investigation. JZ: conceptualization and writing—original draft. Z-HH: conceptualization, project administration, and funding acquisition. WX: conceptualization, writing—original draft, investigation, formal analysis, project administration, and funding acquisition. All authors contributed to the article and approved the submitted version.

## Conflict of interest

The authors declare that the research was conducted in the absence of any commercial or financial relationships that could be construed as a potential conflict of interest.

## Publisher’s note

All claims expressed in this article are solely those of the authors and do not necessarily represent those of their affiliated organizations, or those of the publisher, the editors and the reviewers. Any product that may be evaluated in this article, or claim that may be made by its manufacturer, is not guaranteed or endorsed by the publisher.

## Abbreviations

ABCG1: phospholipid ATP-binding cassette transporter G1
CD36: cluster of differentiation 36
Dil-ox-LDL: oxidized low-density lipoprotein labeled with 1,1 ′ -dioctadecyl-3,3,3 ′ ,3 ′ -tetra-methylindocarbocyanine perchlorate
DMSO: dimethyl sulfoxide
ECL: enhanced chemiluminescence
FBS: fetal bovine serum
HDL: high-density lipoprotein
IL-1 β: interleukin-1 beta
IL-6: interleukin-6
LXR α: liver X receptor α
MFI: mean fluorescence intensity
NO: nitric oxide
ox-LDL: oxidized low-density lipoproteins
PBS: phosphate buffered saline
PPAR γ: peroxisome proliferator-activated receptor γ
PVDF: polyvinylidene difluoride
SRA1: scavenger receptor class A type I
SRB1: scavenger receptor class B type I
TC: total cholesterol
TG: triglycerides
TNF- α: tumor necrosis factor- α.
