# Supplementary material for: Three polymethoxyflavones from the peel of Citrus reticulata “Chachi” inhibits oxidized low-density lipoprotein-induced macrophage-derived foam cell formation
Source: Front Cardiovasc Med. 2022 Jul 28;9:924551. doi: 10.3389/fcvm.2022.924551 (PMC9366847; doi:10.3389/fcvm.2022.924551)
Supplement: Supplementary file 1 [file Table_1.DOCX]

**Supplementary Table 1**

**Table S1. Primer sequences used for RT-qPCR.**

| **Genes** |  | **Primer sequences** |
| --- | --- | --- |
| **IL-1β** | **Forward:** | 5'-CACTACAGGCTCCGAGATGAACAAC-3' |
|  | **Reverse:** | 5'-TGTCGTTGCTTGGTTCTCCTTGTAC-3' |
| **IL-6** | **Forward:** | 5'-CTTCTTGGGACTGATGCTGGTGAC-3' |
|  | **Reverse:** | 5'-AGTGGTATCCTCTGTGAAGTCTCCTC-3' |
| **TNF-α** | **Forward:** | 5'-CGCTCTTCTGTCTACTGAACTTCGG-3' |
|  | **Reverse:** | 5'-GTGGTTTGTGAGTGTGAGGGTCTG-3' |
| **ABCG1** | **Forward:** | 5'-CTGCTGCCTCACCTCACTGTTC-3' |
|  | **Reverse:** | 5'-TCTCGTCTGCCTTCATCCTTCTCC-3' |
| **SRB1** | **Forward:** | 5'-AGCATTCCTTGTTCCTAGACATCCATC-3' |
|  | **Reverse:** | 5'-AACCACAGCAACGGCAGAACTAC-3' |
| **SRA1** | **Forward:** | 5'-GACACTGATAGCTGCTCCGAATCTG-3' |
|  | **Reverse:** | 5'-AAACACGAGGAGGTAAAGGGCAATC-3' |
| **CD36** | **Forward:** | 5'-GTCTATCTACGCTGTGTTCGGATCTG-3' |
|  | **Reverse:** | 5'-TGTCTGGATTCTGGAGGGGTGATG-3' |
| **GAPDH** | **Forward:** | 5'-GGTTGTCTCCTGCGACTTCA-3' |
|  | **Reverse:** | 5'-TGGTCCAGGGTTTCTTACTCC-3' |
